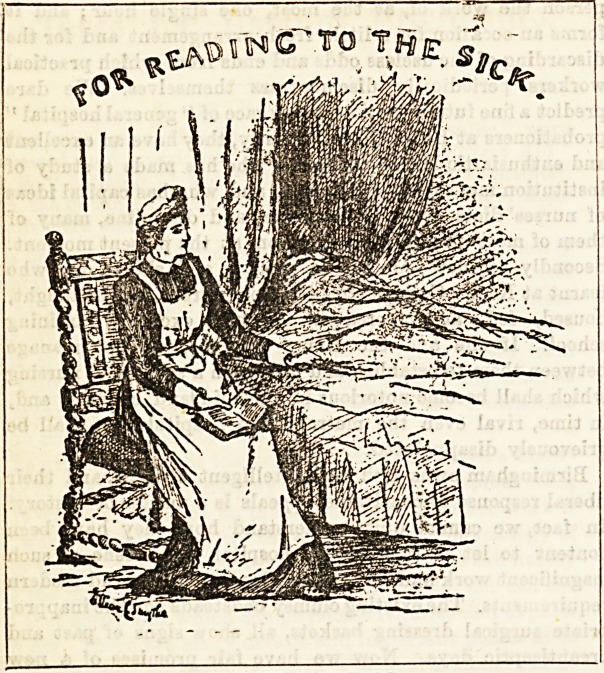# The Hospital Nursing Supplement

**Published:** 1892-08-27

**Authors:** 


### "The Hospital" Nursing Mirror.

Being the Extra Nursing Supplement of "The Hospital" Newspaper.

Contributions for this Supplement should be addressed to the Editor, The Hospital, 140, Strand, London, W.C., and should have the word "Nursing" plainly written in left-hand top corner of the envelope.

### En Passant.

The Derby and Derbyshire Royal Nursing Institution.—The annual meeting of this association was held under the presidency of the Mayor. Its report shows that the year began with £418 and ended with £521 in hand, and the income of the year amounts very nearly to £3,000. Six nurses are now employed exclusively in work amongst the poor, two at the workhouse, and four in the town. Miss Woodhead, the Lady Superintendent, has been compelled to resign owing to the heaviness of the work.

Ayr County Hospital.—Several alterations are forthcoming in this hospital at a cost of £1,500, exclusive of sanitary repairs. Six new bedrooms for the nurses and servants are to be built for the fever wards, and telephone communication is to be made with these wards and the house surgeon's room, and a bath-room is to be added. The nurses and servants in the general hospital will have six new bedrooms, and the nurses will rejoice in a new sitting-room, as the present sitting and dining rooms do not, in the opinion of the managers, afford reasonable comfort for the staff of nurses. Nearly each day we hear of some new consideration and thought for nurses, and nearly every day we hear from grateful and happy women, who recognize that their welfare is anything but forgotten.

The Brassey Holiday Home. — Many of our readers have been to this happy hunting ground this summer, so many will know that Sister Frost and Miss Holditch are obliged to leave their house at St. Leonards, as the landlord is so anxious to live in it himself. The Home is about to migrate to Ventnor, and will be ready in the first week in October to receive visitors. Miss Holditch is taking nurses in at St. Leonards till the second week in September. A lovelier place than Ventnor can scarcely be found, there are so many places to be seen in the island, and the new Home has a sea view, and has a garden with a full-sized tennis-court. The arrangements for the Nurses' Endowed Bed will be the same as usual, and when there is no *sick* nurse using it we shall be glad to give a holiday to nurses who cannot afford for good reasons to take one themselves. We wish every success to a Home where so many nurses have reaped the benefits of rest and happiness.

Short Items.—The Watford District Nurses are paying nearly 8,000 visits; the fund has been working nine years, and adds to its usefulness every year.—The patients of the District Nurse supplied to the Cowley St. John District at Oxford, by the Sarah Acland Home, have subscribed seven pounds to enable the nurse to have more ample enjoyment of her holiday. The nurses are, however, not allowed to receive gifts of money, but the kind thought has been much appreciated.—The Sunderland Board of Guardians have appointed a trained nurse to the post of night nurse.—The ball in aid of the Ryde District Nursing Association was a great success, the Home is already secured, and the proceeds of the ball are to go towards the furnishing.—We hear that already the Darlington Queen's Nurses' Association is about to engage another nurse to work with Nurse Terry.—The ball in aid of the Royal Infirmary at Ryde, was very gay; amongst those present was Miss Fitzpatrick, the matron.

Our Christmas Parcels.—We should like to remind any of our readers who did not see the former notice that we are very much hoping to receive the same number of garments for Christmas distribution as we have had formerly. Anything, from socks to dressing gowns and petticoats, will be most welcome, and so we ask those who have a few spare moments to spend them in stitching for us.

From Poor but Grateful Patients.—Three year's good work has endeared Nurse A. C. Barker, the parish nurse of St. Woollos, Newport, to all the townspeople, and her coming departure is very much regretted. She has received several presents as tokens of esteem and gratitude, amongst them two volumes of "The Life of Dean Burgon," from the Mayor of Newport, and a beautiful dressing-case bearing the inscription "Presented to Nurse Barker from the poor but grateful patients of St. Woollos parish.

Brechin Victoria Nursing Association.—Through the generosity of Miss Duke, of Eskpark, and Mr. Robert Duke, of Bearehill, the Jubilee Nursing Fund of Brechin is to be started; they have given £1,000 to be part of an endowment fund for the provision of nurses for the poor, and have also promised to be subscribers of £10 a year each. In giving this generous donation Mr. Duke quoted the success that had crowned the efforts of those who started district nursing in Dundee, and he predicted a like success for Brechin. A nurse is to be engaged, and she is to start work at once.

The Meath Home of Comfort.—The terms at this home are as follows: Members of the G.F.S., 10s. 6d. a week; non-members, 12s. 6d. a week; children, 8s. a week. Then, in addition to these, a certain number of cases will be received from one to three guineas weekly; a subscription of £4 reduces the payments for the G.F.S., non-members, and children, 2s. a week. Already over a hundred applications have been made at the home, and the committee are hoping that subscriptions and donations will enable them to keep as many free beds for destitute cases as possible. Would-be subscribers should send to Colone Clarke, R.E., Netherton, Guildford, and applications for admission to the home must be made to the Lady Superintendent at the home at Godalming.

Preston Royal Infirmary Annual Festivities.—The season opened on the 21st June by a visit of the nurses of the above institution to the residence of C. R. Jackson, Esq., J.P., to Barton Hall, and on June 27th and 28th, by the kindness of Dr. C. R. Brown, the nurses had an excursion to Fleetwood. After a pleasant sail, they adjourned to the Station Hotel for lunch, and afterwards proceeded to Blackpool, where a most enjoyable afternoon and evening were spent in the Pleasure Gardens and other places of amusement. The annual outing, given by the Matron (Miss Pigott), took place on June 15th to Ormskirk. The whole of the senior nurses left the Royal Infirmary in two stage coaches, and drove through some of the most lovely scenery in Lancashire. After lunch and tea the party returned to Preston, which was reached about 9.30. On July 27th a party of nurses, accompanied by the Matron, at the invitation of Mrs. Pakes, paid a visit to Copp Vicarage, calling on the way at the Lodge, where they were regaled with strawberries and cream by Mr. King. The afternoon was spent at the Vicarage in playing games, tea bringing a delightful day to a close. The year's festivities were concluded on August 11th, by the convalescent patients having a drive and pic-nic in Moor Park. The carriages were lent by Messrs. Harding and Co.

### Lectures for Asylum Attendants.

By William Harding, M.B.

### I.—INTRODUCTORY.

[The members of the class to whom these "talks" were addressed had previously had instruction in "First Aid" and in "Nursing of the Sick." Being already acquainted with the outlines of general anatomy and physiology, these details were omitted.]

It is necessary that the nurse for mental cases should clearly understand that the patients with whom she has to deal are as certainly suffering from bodily disease as are those who are afflicted with an affection of any organ in the chest or abdomen. The mind is not a thing apart from the body. Intelligence is as truly the work of the brain as the movement of the fingers is the result of the contraction of the muscles of the forearm.

The attendant on the insane does not need to have a knowledge of the delicate anatomy of the brain and spinal cord, but it would conduce to an intelligent interest in her work if she were acquainted with the outlines of the physiology of these organs. Without such information the nurse can hardly take the interest in her work that she should do, nor can she give that assistance which the physician requires and expects from her. There are many details and important points as to the habits, &c., of patients, with which she alone can be cognisant, and these may pass unnoticed, if there be no eye trained to note them, and no mind educated to understand their significance.

There is a little creature which consists of but a tiny microscopic speck of jelly-like material. It has no muscles, bones, nor digestive organs as we understand these structures. That one little mass fulfils all the duties necessary for the preservation of life. It takes food into the interior of its substance; absorbs the nourishment there is in it, and puts out the indigestible portions from some part of its circumference. When it wishes to alter its situation, it protrudes a portion of its jelly-like mass, and then, as it were, flows in that direction. Thus the lowest forms of animal life may consist of a simple structureless speck. All creatures higher in the scale are made up of groups of little bodies called "cells." In the lower forms they may be few in number and very simple in structure. As we ascend still higher, we find that animals are composed of myriads of cells, and that certain groups of these have taken particular duties upon themselves, and are changed or modified so as best to perform these duties. Some form a covering, as in the skin; others provide means of movement, as in the muscles; another section aid in the nourishment of the body, as in the digestive tract, or assist in the excretion of harmful matters, as in the kidney. We have here a very different state of affairs from that which prevailed in the one-celled, structureless little creature. Its mass performed alone many duties which in us are undertaken by separate collections of cells, specially modified for that purpose. In order that this complex machine, the body, may be kept healthy and vigorous, it is necessary that these various collections of cells (forming different organs) should be kept in touch with each other, and that there should be some central directing and combining power. To fulfil this end, certain cells were set apart, and these cells form what we call the nervous system. By it the whole body is made to act in harmony; through it the various organs are put in action, or have their activity restrained, as is best for the welfare of the creature.

In the lower animals the nervous system is well developed as regards the needs of material existence; in man it has reached its highest point. By its means he thinks, wills, and feels; thanks to it he is the lord of living creatures.

The cells which form the nervous system are modified into two great classes, viz, nerve cells and nerve fibres. Nerve cells, which are, so to speak, the workshops in which nerve force or brain power, is elaborated, and nerve fibres, by which messages are carried to and from the nerve cells. These nerve cells, embedded in soft tissue, which supports and protects them, are massed together in the central organs of the nervous system, *i.e.*, in the brain and spinal cord. They are of vital importance to the body, inasmuch as they direct and regulate the action of organs upon whose activity life depends, and they are, therefore, carefully shielded from injury by bony cases. The brain is securely placed within the vault of the skull, and the spinal cord, which is continuous with the brain, is sheltered within the cavity formed by the apposition of the various vertebræ.

The nerve fibres are found as fine threads in every part of the body. They join together to form small white cords which we call "the nerves." The nerves of the head pass into the skull directly; those from the body generally run to the spinal cord, which they enter. The fibres pass up the spinal cord, and are connected with nerve cells in it, and also with the nerve cells in the brain. In the spinal cord the fibres are placed superficially and enclose the nerve cells in their centre; in the brain the cells lie chiefly on the surface, which is convoluted or arranged in folds so as to present a large amount of surface when compared with the space occupied. The fibres run into the brain and then spread out in a fan-like fashion, and pass to every part of its circumference.

We thus see that there are masses of nerve cells in the brain and spinal cord which are connected by means of nerve fibres with every part of the body. These fibres are gathered into bundles called "nerves," and for the most part reach the brain through the spinal cord.

We saw that there was a subdivision of labour among the various kinds of cells in the body generally, and that the most important work of all was undertaken by that group of cells which we call the nervous system. Then we noticed that this group was modified into two great classes, nerve cells and nerve fibres. We will find that the duties of the nervous system are so important that they are still further subdivided, and this is the case both as regards nerve cells and nerve fibres.

The nerve fibres are divided into three kinds, which have been named according to their functions. There are fibres which convey messages from the various parts of the body to the brain, and these are called *sensory* or, better, *afferent* fibres (so designated from a Latin word which signifies "carrying towards"). Those fibres which convey messages out from the nerve cells to the body are called *efferent* ("carrying outwards"). Some of the efferent fibres are called motor, because they convey impulses to the muscles. The third kind are called *communicating* fibres, because they run between cells in different parts of the brain and so unite them all.

A nerve may be composed entirely of afferent fibres, and is then called a sensory nerve; or it may be composed almost entirely of efferent fibres to muscles, and is then known as a motor nerve. Some nerves contain both afferent and efferent fibres, and are then called mixed or sensori-motor nerves.

Certain of the purely afferent nerves carry special kinds of messages, as, for example, the nerves connected with the organs of special sense, which have been called the gateways of knowledge. These organs will be dealt with more particularly when we come to speak of hallucinations of sight, hearing, &c. The efferent fibres also are not all of one kind, but consist of fibres which carry orders to muscles, glands, &c.

Various little collections of nerve cells have special duties assigned to them. One of the great functions of the nerve cells in the spinal cord, and especially near its junction with the brain, is to preside over *reflex actions*; that is, actions which can be carried on independently of consciousness, but which are all important to the health and even the life of the individual. These actions are called reflex because the afferent message is not passed on to the brain, but is reflected or bent back by the nerve cell in the spinal cord and the necessary order sent out to the organ or part of the body implicated. As instances of reflex actions, we may mention the closing of the eyelids, swallowing, respiration, secretion of saliva, and micturition. Thus, when food is taken into the mouth the afferent nerve carries the message to the nerve cells, and the efferent nerve conveys a message to the salivary glands to secrete saliva. A person's foot may be tickled, and although he is quite unconscious of it, his foot may be twitched away.

### Nursing Homes.

### BIRMINGHAM GENERAL HOSPITAL.

It covers a great deal of ground, this old-fashioned general hospital, standing in the midst of somewhat dreary and smoke-begrimed surroundings. Certainly it is one of the institutions which was intelligently erected on a spot suitable to its uses, for it stands amidst factories and it is also quite near those busy stations, which, to many a traveller, are the most familiar landmarks in this big and ever-growing town.

The lodge porter promptly answers the gate bell and instructs a visitor to "go across the gravel and up the steps," which lead into a pleasant and quiet hall in the centre of the front wing.

The nurses' home stands in the courtyard behind the main building, and is quite distinct from the hospital. It contains some fifty separate bed-rooms, the usual general sitting-room, and a bath room on each floor. The Sisters have a tiny sitting-room off their wards, but we are glad to find that they all sleep in the nurses' home. The nurses' bed-rooms are sufficiently commodious, and light, and airy, but they form a great contrast in their extreme plainness to those attractive little rooms at the Nightingale Home, King's College Hospital, the London Hospital, and Marylebone Infirmary. Still, it is very pleasant to find the necessity for separate sleeping accommodation for nurses has been thoroughly grasped, in an institution where other modern ideas are lamentably absent.

The Sisters and nurses take all their meals at the hospital, in a plain downstairs' room, which bears a certain resemblance to the dining-hall at the Royal Free, Gray's Inn Road, and occasional cups of tea comprise the only refreshment recognised at the nurses' home.

At present a very objectionable arrangement exists with regard to the night nurses' sleeping arrangements. They occupy the same rooms whether on day or night duty, which means that one apartment is occupied by a probationer who rests during the day, and the next to it belongs to a girl who sleeps at night and works all day. Now what certainty of quiet can there be for the weary night nurse? It is unnatural and, therefore, undesirable, that the day duty probationer should be hushed into absolute silence during her brief recreation hours; and a pleasant peal of laughter or the chattering of young voices should be encouraged at these times, but they are an element of discord when detrimental to the well-being of the night nurses. It is all very well to say that the probationers dislike having to move their things every three months when the change of duty takes place, and that they prefer existing arrangements. As a general rule nurses, like other people, prefer the plan of life which gives to them the least amount of personal trouble. Few of them mind the incessant work which is included in the care of the sick, but they simply hate bothering about their own things; and although we can make allowance for many nurses' whims, this is one which must be quietly laid aside. A quarterly move into a fresh room is to an orderly person the work of, at the most, one single hour; and it forms an occasion for a little fresh arrangement and for the discarding of the useless odds and ends from which practical workers periodically disembarrass themselves. We dare predict a fine future for the coming race of "general hospital" probationers at Birmingham. Firstly, they have an excellent and enthusiastic House Governor, who has made a study of institution management for years, and who has capital ideas of nurses' diets, training, comforts, and discipline, many of them of most practical application at the present moment. Secondly, a new Matron is coming, and she is one who learnt at King's College Hospital how nurses can be taught, housed, fed, and recreated at that excellent training school. If she and the House Governor do not manage between them to establish and maintain a scheme of nursing which shall become notorious in the midland counties and, in time, rival even the metropolitan hospitals, we shall be grievously disappointed.

Birmingham owns rich and intelligent citizens, and their liberal response to charitable appeals is a matter of history. In fact, we cannot quite understand how they have been content to let their valuable hospital, the scene of such magnificent work and experience, lag so far behind modern requirements. The existing clumsy bedsteads and the inappropriate surgical dressing baskets, all show signs of past and preantiseptic days. Now we have fair promises of a new order of things, for a beautiful new hospital has been planned, which is to be erected on a new site in a central position in the town and which will possess all sanitary and other conveniences. It may take four years to complete, and we trust that the electric light, already appropriated as a matter of course in American hospitals, will be equally recognised as a necessity in the new building. In those four years much can be done, for the waiting will not be a period of inaction. Rather should it be a time of swift progress and ambitious striving on the part of all workers, to make the staff as well as the edifice, as nearly as possible perfect. The courtesy of the gate-porter is an index to the pleasant and kindly feeling which pervades this old hospital, and we wish to all future as well as present workers within its walls a prosperous and brilliant success in their undertaking.

### Royal Cornwall Infirmary, Truro.

In aid of the Samaritan Fund established in connection with the above institution a few years ago by the House Surgeon, Mr. E. Rundle, a garden party was held in the Infirmary grounds recently. The weather was beautifully fine, and the large attendance during the afternoon and evening testified to the hold which the fund has obtained on public sympathy. The arrangements for the occasion were mainly carried out by the Matron (Miss Burgess), who had been busily at work for some days seeking to crown the effort with success, and who was the "presiding genius" on Tuesday. She received willing assistance from her efficient staff of nurses and many outsiders, and the gratifying results of their combined efforts will, no doubt, lead to the establishment of a garden party as an annual institution in connection with the Infirmary. Tea laid in the gardens was well patronised during the afternoon, and the Truro Volunteer Band, who gave their services, added much to the enjoyment of the visitors by an excellent programme of music. Many of those present patronised "Aunt Sally," an old friend who had a most energetic director in the person of Mr. Lawrence Carlyon; whilst others listened with pleasure to the concerts given in one of the large rooms of the Infirmary. A sale of useful and fancy articles and flowers was held in the same room. The gardens were decorated with flags, and these, with the pretty pink uniforms of the nurses and the many pretty gowns of the visitors, made a very gay sight. The patients, who had been regaled with a sumptuous tea of fruit, cream, and cakes, watched the proceedings with great interest from the windows.

### SLEEPLESSNESS.

How delightful it is to go to bed with a pleasant weariness about us, fall asleep at once, and after a long dreamless nap awake in the morning feeling strong and refreshed for the duties of another day. Age and sickness, however, change our experience of night. The old sleep but lightly and "rise up at the voice of the bird," and those who are suffering find darkness only brings fresh troubles. Wider and wider awake do we grow as the hours creep slowly on, and the slumber we court flies from our wooing. We become victims to all sorts of terrors and fears, which are worse even than pain, and our minds get filled with sad, murmuring, unloving, and discontented thoughts. We try all sorts of devices to pass away the time. We count sheep passing through a gate, we recall poetry we have learnt years before, but just as we seem dropping into a doze a sudden twinge of pain in a moment undoes our efforts, and we groan out with the rebellious Jews, "Would God that it were morning." Alas! when the morning comes after our restless tossings we are tempted again to cry, "Would God that it were evening."

For this, which is among the saddest trials of sickness, we have but to be patient. If we can pray to God to help us He will come to our aid and send the "poppied dew of sleep" upon our eyes in His good time, but if not, He will give such happy thoughts as shall make us feel we are in His care, that He watches over us and makes all our beds in our sickness. The patient Job dreaded the coming of night as much as we do. In his calamities he complained, "Wearisome nights are appointed me"; but at the same time he grasped the truth that they did not come by chance, but were sent by One who knows what is for our welfare. Out of the darkness of sorrow comes the light; our truest comfort is that God has appointed our sorrows, and will smooth our restless and uneasy couch with His loving hand. Our poor weak brains, frightened by fancies and sights which it has itself conjured up, will be soothed and reassured by this thought. No, try whatever source we may, we must come back in the end to meeting our trials in our wearisome nights, as appointed for us by God.

Our blessed Lord's night of agony was appointed for Him that He should be made perfect through suffering, and doubtless we, too, are being strengthened by the angel which came to Him in His agony. Only we must pray, as He did, with all the faith we can summon, and if our aching limbs and restless bodies show the Almighty is trying us to the uttermost, we will endure, knowing that though the night be dark and dreary, joy cometh in the morning.

### Everybody's Opinion.

*[Correspondence on all subjects is invited, but we cannot in any way be responsible for the opinions expressed by our correspondents. No communications can be entertained if the name and address of the correspondent is not given, or unless one side of the paper only be written on.]*

### A HINT FOR A HOLIDAY.

"One who is Interested" writes: To those happy mortals who have not yet had their summer vacation, with a fortnight at their disposal, I would recommend the trip which I myself took in the early part of June, by one of the Clyde Shipping Company's boats from London, via the west coast of England, calling at Waterford or other of the Irish ports, to Glasgow, spending a few days in Scotland, and returning by the east coast from either Grangemouth, Granton, or Leith at option by Carron Line, General Steam Navigation Company, or London and Edinburgh Shipping Company, respectively, the cabin fare for this circular tour being £2 7s. 6d. (inclusive of steward's fee), available for one year. The living on board does not cost more than 7s. per day. Leaving Irongate Wharf, St. Katharine's Dock at noon on Thursday, the 9th, and steaming down the ever-interesting water highway, passing the numerous docks, Greenwich and its magnificent hospital, Tilbury, and the Nore lightship, we get fairly to sea, and when retiring to my snug bunk have the lights of Eastbourne well in sight. On awaking next morning we are moored alongside Southampton Quay, and are able to spend some hours ashore. We reach Plymouth at eleven the same evening, and remain until three o'clock Saturday afternoon to discharge cargo and load other. From Plymouth to Waterford, where we arrived Sunday afternoon, we had a rough time of it. The three channels meeting here render it pretty stiff at all times, and on this occasion prevented many from quitting their berths all day, so troubled were they with *mal-ae-mer*. The boat sailed from here on Monday, the 13th, and arrived in Glasgow on Tuesday afternoon. Staying here, walking the length of Loch Lomond, crossing to Inversnaid, and making my way by Loch Katrine, through the Trossachs to Callander, Donne, Dunblane, Stirling, to Edinburgh, I re-embarked at Granton on Wednesday afternoon, and reached London seven o'clock Thursday evening after a bracing and enjoyable holiday.

### ANOTHER HINT FOR A HOLIDAY.

"A Nurse in Ireland" writes: The beautiful sunshiny weather we have been enjoying in the South of Ireland, together with the magnificent scenery which greets you on all sides, have made my thoughts turn to those nurses who have not yet had holidays, and who are in search of a locality in which to spend them. The neighbourhood of Cork posseses in an eminent degree advantages superior to those of many other places. The harbour is one whose natural attractions are unequalled, and for a few pence you can, in a good river steamer, enjoy views of country which cannot be surpassed. For the small sum of 1s. 8d. the steamer takes you first-class from Cork to Crosshaven and back, a distance of some twelve or fifteen miles. Arrived at Crosshaven, you leave the river, and a walk of a mile brings you in full view of the Atlantic, and not only are you charmed by the "water," but lovely country surrounds you, hills on all sides, ferns and flowers. No happier spot could be found in which a weary toiler may regain fresh life and strength.

### A WARNING.

"Master Mariner" writes: I venture to suggest you should advise trained nurses when agreeing to take charge of patients going out to the colonies that they should insist on having a first-class return ticket taken out in their names, or they may find themselves very much out of place with only a servant's ticket to bring them home again. Also that they should have an understanding what provision is to be made for them in the colony from the time they arrive till the next opportunity of returning. I write this as I recently came across a nurse who had been left to shift for herself on arrival, and on presenting herself for conveyance home in the ship I found she had only a servant's ticket. Fortunately the ship was not full, so the good lady was not inconvenienced as she might have been.

### MASSAGE.

"MASSEUSE" writes: I feel I must write you how pleased I was to read your little article on "Massage" in the "Nursing Mirror"; I quite agree in all you say, and I prefer to work under the supervision of a medical man; I have had a private practice nearly four years, and find I still have much to learn, which can only be acquired by experience and study, and daily practice; and I find the HOSPITAL a great help to me, and take this opportunity to thank you for your useful paper.

### Appointments.

INGHAM INFIRMARY, SOUTH SHIELDS. — Miss Ethel Rimington, who has been holding the appointment of Head Sister at this infirmary for the last few months, has been elected Matron on the resignation of Mrs. Brewis, who has resigned after eight and a half years' work. Miss Rimington's appointment has given great satisfaction.

HOME FOR INCURABLES, STRATHCLYDE HOUSE, CARLISLE.—Miss A. M'Bryde Brown, late Sister at the Leicester Infirmary, has been appointed Matron of the above institution. Miss Brown was trained at the Children's Infirmary, Liverpool, and was afterwards staff nurse at King's College Hospital, London.

### Notes and Queries.

### Answers.

*Medicus.*—We cannot prescribe. We are constantly mentioning this fact. You ought to ask a medical man, for such excessive perspiration must be the result of ill-health. Go to your Matron and tell her you want to find out how to get rid of it, and she will doubtless help you.

*A. J. M.*—Many thanks for kind note; it is, we assure you, very encouraging to meet with gratiutde.

*A Nurse in India.*—We much regret that a letter signed only as above comes to us by this mail, commenting on the prospects of nursing for India, but we cannot publish it as our correspondent breaks a rule to which we must adhere firmly, viz., that address and full name must be given as a guarantee of the good faith of our correspondents.

*Norbiton.*—We are procuring full information for you, and will write you every particular.

*A Home in Scarborough.*—In answer to the query in THE HOSPITAL for August 20th respecting a Home for Nurses at Scarborough, I once stayed at 4, Esplanade Gardens, Scarborough, where I was most comfortable, and spent a most enjoyable holiday for a week alone. The next week a friend kept me company. We paid £1 a week for one person and £1 10s. for two, and had boarding and lodging. Miss Empringham told me she would always take nurses for the above charge. We had a very comfortable bedroom, and the use of a sitting room, but we were out from early morn till bed-time, excepting for meals. These were always ready for us at the time we asked, and we had no trouble about food, and bringing it in, &c., as we had all we could desire provided for us. I do not know of a home for nurses, but possibly this will answer the purpose, and I feel sure Miss Empringham will do her best to make others as comfortable as she did me. The house has the additional advantage of being close to the sea, &c.—*E.M.S.*

### Wants and Workers.

There are several pressing wants the last few weeks which we insert, hoping some of our kind readers can either help or tell their friends of these small corners in the land of sick folk which want filling badly.

*The Lady Superintendent, Sanatorivm, Bromsgrove,* would be greatly obliged for a carrying chair, of which the institution is in great need; also for crochet or woollen shawls for the patients breakfasting in bed.

Wanted, a home for an orphan girl (24), requiring firm, kind treatment. Willing to do light housework under supervision, and able to pay 8s. weekly. Address, with full particulars, *Sister Lucie, Littleborough, Lancashire.*

*Sister Katherine, St. Mary's Nurses' Home, Plaistow, E.,* will be glad to know of a home, free, for two little girls 7 and 8, whose mother has terrible fits, and whose step-father illtreats them.

*Home for an Epileptic.*—Can anybody tell us of a home for a young girl, no hope of cure now, a patient in an infirmary. Five pounds a year could be paid for her and her clothing found; she is able to assist generally when free from fits.

*The Matron of the Ledbury Cottage Hospital* will be very grateful for an old piano for the patients. Will our readers ask among their friends.

### Some Australian Experiences.

(*Continued from page* lxxxiv.)

MY second charge was an experience with a vengeance, although there was a ludicrous side to it, which I did not see at first. The patient was an Englishwoman who had come out to join her husband, who held a fairly good position near Sydney, bringing with her six children, the youngest of whom was nine months. This unfortunate woman contracted enteric fever at one of the ports, and when she landed was very ill, yet she took an empty house, to which she brought her children, luggage, and a few beds to await the arrival of her husband. The poor creature struggled on for a few days, and then called in a doctor, who found her on a palliasse on an iron bedstead, with no mosquito net, or any semblance of comfort about her, so he fetched a mosquito net from his own house, prescribed for her, then came and asked me to go and see to her. Fortunately the attack was a mild one, and after sponging and making her comfortable, her temperature did not rise again above 102. The fact of her being in a delicate condition at the time did not improve matters, as you may easily suppose. What about the poor children you will say? I lack power to describe their condition. They were fearfully dirty, and covered with mosquito bites. By scratching these they had brought on a specie of "glass pock," commonly called "native pock." The poor things were objects; four out of the six were quite helpless, and the poor baby was just the age to be afraid of a stranger. The two eldest boys were so fully persuaded that they were just the two boys Australia was waiting for, that they spent most of their time answering advertisements, and I got very little help. After a few days' fruitless trips to town, they began to see there were other boys able to read and write as well as themselves, so they stayed and chopped wood for me. To go back to my first evening, as soon as the mother was as comfortable as circumstances would allow, I turned my attention to the children. I borrowed a large washing tub and set to work to give them all a good "bogie" (native term for bath), and put them to their wretched apologies for beds, for they were simply beds on the floor, and the covering consisted of all sorts of garments. Certainly they did not so much require covers for warmth as for a protection from mosquitoes. Our cooking was as primitive as our bathing; a frying-pan and kettle formed the kitchen utensils, and all sorts of odd crockery, &c., formed the dinner service. We all managed somehow, the mother in the meantime steadily improving. We persevered in our "bogie" every night, and I fancy I must have presented a similar picture to "Mark Tapley" when he was washing the emigrants' children. The only rest I got was on the edge of my patient's bed until the doctor interfered, and found a room for me at a cottage near; he also hunted up a "washer lady," who consented to come so long as she was sure the patient would not die and she have to lay her out. She turned out a very nice old body, and was the means of my escape at the end of a fortnight, when the husband appeared on the scene. It was an anxious time, the patient, was so fretful at the idea of getting ill so soon, and the poor baby, who was cutting its teeth I had to take for long walks in order to enable her mother to sleep. For meals, we had incessant fried tough steak, &c., and I was not sorry when the doctor pronounced my patient safe to leave. The doctor was the only one who showed any gratitude for what I did; the patient was greatly afraid she was not getting quite enough out of me. She did not understand all I had had to do, but the fact that I got her through was sufficient for me, so I left my first English case without a spark of regret.

I will not tire you by entering into all the cases I nursed during the next six months, but suffice it that I never got another unpleasant one; on the contrary, they were all amongst the kindest of people, all Australians, and twice during that time I was asked to make my home with them in between my cases, offers I had reason to regret having refused. The great kindness I received I shall never forget. I found the Australians brimming over with geniality and hospitality, for although in many cases one had to "buckle to" and give a hand in everything, at no time was it ever taken as a matter of course, except by my English patient already mentioned.

After nursing in and about the capital all the summer, I was glad, after a short holiday, which I spent at the famous Blue Mountains, about fifty miles from Sydney, to accept an engagement to go "up country" to nurse a squatter's wife, in one of the largest squatting districts in New South Wales, known as the "Riverina," about a thousand miles from Sydney. I took the night mail train to a place called "Junee," where a branch line leads up to the Plains. I had to spend part of the night at the railway station, which is most comfortably fitted up as an hotel as well. Of course, being strange, I imagined all sorts of horrors would happen on my first experience of a night in the country, so as a small precaution, I pushed a chest of drawers across the door, for fear of sudden invasion, and fell asleep to dream of bushrangers and places being "stuck up" by these interesting gangs. The "Kelly Gang" had only a few years previously been captured, but I am glad to say I awoke to find myself quite safe, and a very comfortable breakfast awaiting me below. I then resumed my journey, but was astonished to find a large number of policemen occupying the best part of the train; upon inquiry, I found they were on their way to different "stations" on account of an arising difficulty with shearers (I say arising, as it took some years to develop into the great strike). It was a most tiring, uninteresting journey, across miles and miles of flat country. Now and then we passed at a distance, a clump of trees, then a lot of sheep—for this is a sheep country—then away, away we went across more plains, occasionally starting a few emus who scudded across the track, frightened by the train; not the sign of a homestead all the way. There are small landing stages along the route—merely a rough platform to alight upon, and these, as a rule, are some miles from the dwellings, so folks are fetched in buggies, &c. I was very much astonished to see some members of the "Salvation Army" at one of these places, they had come to greet a "lassie" on her way "up country." It so happened the "plains" were at their best, and covered with wild flowers, marsh mallow, and everlasting flowers; they came as quite a relief to an otherwise tedious journey. Long after sunset we arrived at our platform, and the guard came to tell me it was my place to alight (I had made this particular journey somewhat lively by worrying the poor man, lest he should forget me, and it must have been quite a relief to him when I left). A very pleasant, rosy-cheeked lad, who looked like the "curly-headed plough-boy," came up to me and said, "I am waiting for you, Miss." When I turned to follow, I found a large body of police who were *en route* for the same "station" as I was. Out on the track buggies were ready to convey us to the "Homestead" as the head quarters of a "station" is called, and after a long drive we arrived at our destination. Of course, in these back country places the arrival of any sort of stranger causes a small excitement; such important people as myself and the police was more so, so the first thing I saw was a group of children peering out to see the arrivals. I was surprised to find such a lovely dwelling in an apparent wilderness, for I was shown into a sort of general sitting-room, furnished precisely the same as in town, where my future patient came in to welcome her nurse. She was most kind, and after enquiries as to the journey, tea was sent in, after which I was shown to my room to rest and get off my dusty things. This done, I went in to supper, and the talk of work was postponed till morning.

*(To be concluded.)*

### Our Holidays.

Although work may fill the chief part of our lives, the occasional holidays in which we indulge fill no inconsiderable portion of our thoughts. Unconsciously we date things as having happened before or after "I went for my holiday," and when that event has passed into the remoteness of "last year," we begin to anticipate the next annual outing.

It is of nurses that we propose to talk just now, and we will begin with the leave to which matrons are entitled. When trained superintendents of nursing first began to form schools for the teaching of probationers, they often found committees very hard to convince that regular holidays for each member of the staff must be an accepted fact—a right, and not a favour.

Some of these gentlemen who have recently held up their hands and exclaimed with pious horror at the poor probationers, getting only a fortnight's summer holiday, seem conveniently to forget that it was they and their contemporaries who were with difficulty persuaded to grant even these two weeks. Long accustomed to deal with a class of women who had neither demanded nor desired legislation on a subject of little personal interest in their own lives, these gentlemen would not grasp the fact, plainly presented to their notice, that with the new order of intelligent and skilled nurses, improved arrangements were needed. The old, poor, and ignorant nurses could seldom afford to go any distance away, for travelling was expensive in those days and wages were low, and the diet provided was so insufficient as to need supplementing in various details, which taxed heavily their scanty earnings. But the superintendents who had, with pains and patience, acquired such experience as justified them in demanding similar knowledge from their subordinates, made a brave attempt, in the face of the discouragement given by old-fashioned medical officers and committees of management, to give to these new probationers whom they proposed to train fittingly not only regular hours of work but regular days of play.

If the nurses of to-day could look back, as we do, instead of grumbling at the present, they would hold shamed silence in remembering those days in the past. Not a vague and shadowy past age, but a period clearly recollected by every thoughtful middle-aged man and woman.

The matron or superintendent was generally granted an annual holiday without much demur, but her making use of the permission was quite another matter. Evolving order out of chaos was a herculean labour, needing days and nights of unceasing toil and anxiety. Moreover, that delightful class of onlooker to whom "I told you so" is the most congenial of refrains, was always on the watch for any mishap or for any irregular proceeding which could be, fairly or unfairly, set down to the discredit of the new system.

Therefore, the granted holidays were often either shortened or allowed to lapse altogether by the anxious and ambitious matron. Her reward came to her later, when she had succeeded in forming and setting in motion such a practical scheme of skilled nursing as has really formed the foundation of those schools of training in which all England takes pride. Now that the machinery works with the smoothness begotten of habitude, both matrons and their assistants take their well-earned holidays regularly. Some have the month of July or August, when the lectures to probationers are discontinued for the few weeks succeeding the annual examinations on technical subjects, an event fraught with many anxieties to the young aspirants for fame. A liberal-minded committee or board will also generally advise that an additional week shall be offered to the matron once or twice during the year, at such seasons as have been especially heavy in their demand on her personal administrative resources. In cottage hospitals at least four consecutive weeks should be granted to every matron; her work is generally fairly incessant, and is accompanied by the strain of unshared responsibility.

Sisters' holidays vary considerably in hospitals, for whilst one place gives only three weeks at a time, it may be the custom to allow extra days if specially desired, and the monthly Saturday to Monday absences, which go far to maintain the health of the workers.

Another hospital gives four weeks, and a monthly day off, but objects to the coveted night being spent outside the precincts. This is short-sighted policy, for a day's rest is doubled in value when sister or nurse is permitted to depart to her friends at seven or eight o'clock one evening, returning next day at ten p.m. refreshed and vigorous, to resume her congenial but arduous ward life.

Probationers will ere long be, universally, we trust, treated as they already are in one or two favoured institutions, where one clear month is granted to them after their first twelve months of training. It certainly pays in the long run to rest, as well as to feed, our young nurses, well, and reasonably. In the meantime they should give their whole minds and strength to their work whilst they are engaged in it; not letting their thoughts stray from their duties to selfish considerations of how much extra "time off" they can contrive to secure for themselves. Fair holidays annually, and honest days off duty; not days which have commenced in a ward, sending the worker to her twelve hours of freedom, heated and wearied by three hours' arduous labour, which take the edge off any girl's appetite for the natural recreation which she has earned already. Again, the extra holidays, known as sick leave, vary considerably in different places. We know of only a few hospitals, and one or two infirmaries, where the wise system prevails of encouraging workers to take a quiet day or two's rest when they need it. Few good nurses are wishful to claim this indulgence unnecessarily, but the knowledge that it is never grudged to them is valuable to a degree which only one of themselves can estimate. Of course most hospitals grant a certain amount of sick leave without detriment to the probationer's prospects, but when it is given unwillingly, when the ailing woman is allowed to see that her weakness is considered troublesome, she, for her part, dislikes to mention the small ailments which make work, temporarily, a weariness. It is very bad when useful women are constrained to conceal their craving for a little extra rest or indulgence, because they are discouraged from owning up to their feelings. Bracing is mentally and physically wholesome, and we could never wish to encourage "nerves" or "fancies"; but good earnest women soon set these aside in the realities of sick nursing, and we owe it to them to help to conserve the strength which they are generally willing to expend most cheerfully in the service of the needy.

### Death in our Ranks.

We much regret to announce the death, on the 9th inst., of Sister Maud Mausel, of Her Majesty's Indian Nursing Service, from cholera. Miss Mausel was the youngest daughter of Robert Shum Mausel, J.P., formerly of Rothbury, Northumberland. (By telegram).

### "The Flowers of Sleep."

Gilbert Carew, the new assistant master, stood a moment outside the building where the central classes were held, before opening the door. These classes have been lately established in most large towns for the purpose of giving instruction to the Board School pupil teachers. It was early, about half-past eight o'clock, and the first day of the Easter term.

He was a tall, slight young man, with stooping shoulders, and a thin and somewhat drawn face; his hair was light, his mouth lacked decision, his blue eyes were keen and vivid.

He was making a few resolutions before entering on his new career. "It shall be a struggle to the death," he said to himself. "I will wrestle with all my weakness, all my hesitation, all my lack of imposing discipline, as if I were a monk of old striving to overcome the Devil. I will give up reading my books and writing my poems, and devote myself body and soul to the work I have undertaken." And quoting to himself Browning's lines, "I was always a fighter, so one fight more, the best and the last," he pushed open the door.

The superintendent of the classes, a short, upright man, was already seated at his desk. "Mr. Carew, I think?" he said, rising, "I am glad you have come early. There are one or two points I want to talk over with you. First of all, have you found comfortable lodgings."

"Thank you, yes. I have acquired a couple of nice rooms in Marion Square."

"That is satisfactory. And now to business. You are not yet practically acquainted with the workings of the central class system."

"I have studied the last code and the directions to Government inspectors; and may I ask what point the students have reached in history?"

"O, begin at the beginning. The last man we had (1) taught them nothing but the dates connected with the Roman occupation."

"And a firm grasp of early history is so essential to a just comprehension of its complex developments."

"What books have you been using?"

"None in particular. I read up all the important authorities I could procure."

The Superintendent looked at him with a slight lift of the brows. The assistant master was certainly out of the common, and new blood was all very well in its way, but would he be able to teach facts and to maintain discipline? However, Atkins only remarked, "Perhaps you would like to take down the names of the students."

While he was engaged in this occupation, a young girl came into the room carrying a large pot of primroses she had been arranging. She was small and slight with dark hair; her pretty oval face and delicate features were set off by a terracotta coloured dress.

"Good morning, Mr. Atkins," she said, shaking hands.

"What early primroses, Miss Pierce! Did you gather them yourself?

"Yes, in Bramley Woods," she replied. "I came down from London on Saturday, and spent yesterday out of doors. By the way, will you give me another ticket for the Pupil Teacher Concert and Prize Distribution next month? I want to bring my friend with me."

"Certainly," replied Mr. Atkins, going to his desk. "Oh, may I introduce our new colleague, Mr. Carew, to you? Mr. Carew——Miss Pierce. Miss Pierce is our mathematics and science mistress," he explained.

Miss Pierce thought the pale young man's appearance very interesting. He seemed to her typical of the student and of the dreamer. There was but little time for conversation, however, for the pupil teachers were beginning to arrive.

An hour later Carew stood before a mixed class of first year students—boys and girls about fifteen years old—to give them a lesson on "The English in their Continental Home." It was a subject with which he was very familiar, and he began with an able account of the civil organisation of the Saxons, and the basis on which the community was founded. He touched lightly on the difference between "thegn" and "corl," and was about to enumerate the characteristics of early English civilisation, when he became suddenly aware of a look of blank dismay on the faces of the class. He pulled himself up immediately and commenced asking questions, which revealed to him the fact that the class had not understood a word of his lesson. Feeling that he was losing his hold, Carew tried to awaken interest by describing the early art of the English—their buckles of curious and exquisite form set with rough jewels; their arms, the long sword, the shield of linden-wood—and there was a flash of enthusiasm when he recited from "Beowulf," lines "short, sharp-sounding, each like a sword-blow," and wrote examples of Runic letters on the board. But it was only for a moment. Living as the facts were to himself, he had no power of making them live for others; his statements were rather argumentative than dogmatic; the class floundered for some time vainly after him, the sense of their mental confusion came upon him with overpowering force, and he forgot to notice that John and Thomas were whispering together in the back row. Now and again he fixed his eyes on the primroses as if to gather fresh strength from them, but they only breathed through all his senses a wild longing to escape all this futile strain, and plunge into the rich-smelling, sunlit glades of some lonely wood.

"I think you will like Mr. Atkins, the Superintendent of the Central Classes," said Violet Pierce, to her friend, Rachel McClure, a teacher at the Girls' High School. They were walking to the Town Hall, where the prize distribution and concert were to be held. "He is so clear-headed and energetic; his lessons are most interesting—not that he knows very much, he is only learning himself—that is perhaps why he is so enthusiastic, and why he puts everything in such a vivid way that you never forget it. Of course, he is extremely popular with both girls and boys."

"Yes, I shall be glad to see him. I hope the English Master of whom you speak so much will be there too," said Rachel. She was well built, with pale face, level brows, and dull brown hair, drawn to a simple knot at the back. She wore a grey dress, her straw hat was trimmed with artificial poppies, and a few real poppies were stuck in her belt.

"Mr. Carew? He is sure to be there," replied Violet. "I am afraid he is overworking himself. He looks paler and more worried than ever."

"It must be a very hard life. I often wonder how you stand it."

"I love my work better than anything else in the world, you know," said Violet simply.

"Well, this Mr. Carew. Is he also popular?"

"No," answered Violet, "I cannot imagine how it is, for he takes immense pains with his lessons, and you would think his personality would be attractive, even to pupils so young. But he is too far beyond them. He cannot descend to their prosaic level, nor they reach his intellectual height, and so, as a teacher, he fails."

"How sad," said Rachel, in a low voice.

"It is heartrending," replied Violet. "I often wish I could help him, or even show the great sympathy I feel, but he seems morbidly conscious of his failure, and keeps entirely apart. I know he spends whole evenings preparing lessons, which he sometimes finds it almost impossible to give, owing to the disorder of the class."

They had by this time entered the hall. Mr. Atkins and several members of the School Board were seated on the platform. Most of the Assistant Mistresses and Masters were in the front seats, but Carew and the Drawing-master were leaning against the wall for the room was full.

Rachel obtained a chair in the fourth row. The Superintendent delivered a sensible and humorous speech, and Rachel keenly appreciated its many telling points. There was perhaps a little too much of the ego in it, she thought, but the circumstances made this almost inevitable, and his buoyancy and evident unconsciousness acquitted him of all intentional obtrusiness.

Then followed the prize distribution and some glees by the pupil teachers.

Carew remained leaning against the wall. His eyes wandered over the room. A vivid bit of colour—the red of the poppies against the grey of the gown—caught his eye. Just as the primroses in the class-room had struck him by reason of contrast with the hard symmetry of desk and dull vacancy of wall, so the simple form and glowing hue of the flowers attracted his attention as they shone amid crude colouring of dresses and awkward drapery. Then he looked at the girl who was wearing them. What a calm, intellectual expression on the pale face! She seemed to him above those who were round her; like one who has solved the riddle of life, and risen beyond our aimless turmoil. As he looked at her deep and noble eyes and the serenity of her expression a new hope was born within him, and the world ceased for the moment to be dark and gloomy.

"Mr. Carew," said Miss Pierce, in her bright, bird-like way, coming up to him in one of the pauses, "will you not sit down? You will be so tired! There is a chair vacant beside me."

"Thank you very much, Miss Pierce, I would rather stand and watch the people."

Violet looked a little hurt at his reply; she hesitated a moment whether to speak to him again, and then went quietly back to her seat.

Rachel followed her friend's movements. She was not struck by the lanky young man leaning awkwardly against the wall.

After the entertainment Violet introduced Miss McClure to Mr. Atkins. Rachel complimented him on the success of the evening, and remarked on the attractive appearance of the pupil teachers.

"They have so much more individuality than my girls," she said.

"Because many of them know already what life is," he replied. He had a frank pleasant way of speaking that made her feel she had known him a long time. "You would hardly believe what kind of homes some of these students come from."

"Yours is a noble work," said Rachel, "your opportunities are very great."

"So is our responsibility," he answered smilingly, "and we are never free from it, for the students are dependent upon us not only for their instruction but for their amusement also. We have just hired a fine cricket ground for the boys."

"And what have you done for the girls?"

"We are organising a tennis club. The Master of the D—— Street Board School has kindly lent his playground to us after school hours. We open next Saturday."

"With games of tennis, I suppose, and conversation, and tea?"

"The tea must come later; we cannot manage that just now," the Superintendent replied, "our funds have all gone on nets, and bats, and balls."

"Will you let me help you with the tea?" asked Rachel, blushing deeply, "I should like so very much to take part in this work, and to get to know your girls."

"You are very kind; in the name of the pupil teachers I accept most gratefully." His unexaggerated reply put her entirely at her ease, yet she felt that a very genuine appreciation of her proposal was implied in his tone and manner.

"It is settled, then," she said, "I will get all particulars from Violet. Good night."

"Good night: I look forward to meeting you again," he replied.

*(To be continued.)*

## Figures and Tables

**Figure f1:**